# Gender Differences in Psychological Strengths Among Rural Adolescents

**DOI:** 10.1111/jcap.70048

**Published:** 2025-12-15

**Authors:** Angela J. Preston, Jodi Bullard, Rachel Smith, Ashlyn Peppler

**Affiliations:** ^1^ University of Texas at Tyler Tyler TX USA

**Keywords:** behavioral health, gratitude, hope, mental health, optimism, positive psychology, psychological capital, resilience, rural health, self‐efficacy

## Abstract

**Purpose:**

Rural adolescents often lack access to mental health care along with increased rates of depression and suicide completion. While psychological strengths have been associated with positive outcomes globally, little is known regarding the prevalence of these indicators (hope, self‐efficacy, resilience, optimism, and gratitude) among adolescents living in rural Texas. The purpose of this study was to describe how rural adolescents report their psychological strengths and examine the data for potential gender differences.

**Design and Methods:**

A secondary analysis of cross‐sectional data was performed on the RStudio platform using tests of central tendency and inferential statistics.

**Results:**

There were 425 valid responses. Rural adolescents possessed indicators of psychological strengths at varying levels, with 16.9% reporting high resilience and 9.2% reporting high optimism. Males reported significantly greater levels of hope, self‐efficacy, resilience, and gratitude with gender effect sizes ranging from 2% to 5%.

**Practice Implications:**

Within school and community settings, nurses and adults who interact with rural adolescents should consider incorporating tailored strategies that promote growth of psychological strengths, with particular consideration for targeted screening and interventions for at‐risk groups.

## 1 Background

One sixth (16%) of the world's population is comprised of adolescents, defined as individuals between the ages of 10 and 19 (World Health Organization 2024, 2025). The United States (U.S.) adolescent population is slightly less, with 42 million adolescents comprising 12.8% of the overall population (United States Census Bureau 2017, 2020); their representation in the total U.S. population is expected to fall to 11.1% by 2050 (United States Census Bureau 2017). Fewer young adults percentagewise will be required to support society, a phenomenon which presents profound challenges in politics, culture, and socio‐economic norms (Mohd Tohit & Haque 2024). Adolescent mental wellbeing is significant in that they need to be mentally well to positively contribute and fill the gaps that the aging population is leaving behind (Barry et al. 2024). Equipped with this information promotive healthcare measures not only focused on the physical well‐being, but also the mental well‐being, of future generations of adults is of importance to all involved (Mohd Tohit & Haque 2024).

Physical health interventions to prevent illness before it occurs have been developing for over 200 years, with the publishing of Jenner's smallpox inoculation findings in the late 1700s serving as a catalyst (Kayser and Ramzan 2021). Meanwhile, a focus on health promotion for mental health has lagged, with Beer bringing attention to mental health promotion over 100 years later in the early 1900s (Singh et al. 2022). However, with mental illness impacting more than one in five U.S. adults, half of mental health disorders starting by age 14, and 75% of mental health disorders starting before age 25 (Colizzi et al. 2020; McGorry and Mei 2018; National Alliance on Mental Illness 2024, n.d.), promotive mental health interventions at a young age are as relevant as childhood vaccines are to preventive physical health interventions.

### 1.1 The Adolescent Period

Adolescence is a period of significant changes physically, emotionally, and socially (Best and Ban 2021). During this time, rapid brain growth takes place and is influenced by the quality of the surrounding environment which significantly impacts brain development and the potential for mental illness (World Health Organization 2025). The environment an adolescent is immersed and raised in can contribute positively or negatively to their overall childhood experience, thus influencing their mental health over the course of their life (Centers for Disease Control and Prevention 2024). Adverse Childhood Experiences (ACEs) are defined as potentially traumatizing events which occur from birth to age 17, and 75% of high school students state they have experienced one or more ACEs (Centers for Disease Control and Prevention 2024). These traumatic events can include, but are not limited to, experiences of abuse or neglect, witnessing violent acts in the home or community, and emotional trauma (Centers for Disease Control and Prevention 2024; Hughes et al. 2017). The incidence of mental health disorders in the adolescent population is steadily increasing (Agency for Healthcare Research and Quality 2022; National Institute of Mental Health 2024). Therefore, a focus on fostering intrinsic coping mechanisms for times when ACEs are unavoidable are needed (Centers for Disease Control and Prevention 2024; Ortiz 2019).

### 1.2 The Positive Psychology Paradigm

A mid‐twentieth century paradigm shift changed the scope of mental health care from one historically focused on positivity and nurturing to one of pathology and disorders, portraying patients as victims of mental illness (Seligman 2002). In 2002, Seligman's sentinel work on positive psychology focused on reversing this mental healthcare trend by utilizing patients' inherent strengths to assist them in weathering negative life events that have the potential of leading to mental illness (Seligman 2002). Positive psychological strengths have been shown to moderate negative psychological experiences in adult and youth populations (Hinojosa and Hinojosa 2024).

A few developable positive psychological strengths include: hope, a belief or perception that one's goals are attainable – big or small (Snyder et al. 1997); self‐efficacy, the ability to view oneself as an agent and driver for action (Gambin and Święcicka 2012); resilience, the ability of an individual to cope and adapt to changing situations (Connor and Davidson 2003); optimism, a self‐regulatory mechanism that favors problem‐focused coping strategies and faces adversity with an expectancy of success (Scheier et al. 1994); and gratitude, a moral sense of appreciation for the good in life even when faced with or following a stressful event (Froh et al. 2011). The combination of hope, self‐efficacy, resilience, and optimism have been coined as psychological capital, and are frequently referred to as *HERO* strengths (Finch et al. 2023; Luthans et al. 2007). Gratitude is not distinctly considered an aspect of psychological capital but is a closely related psychological construct associated with more positive life experiences (Preston et al. 2023). Psychological strengths, such as the *HERO* strengths and gratitude, are associated with improved mental and behavioral health outcomes in the studies that have been conducted with youth (Preston et al. 2023). Overall, positive psychology is about assisting individuals with building buffers to weather difficult experiences by nurturing their strengths rather than correcting their weaknesses (Seligman 2002).

### 1.3 Rural Adolescents at Heightened Risk

Certain living environments and physiological characteristics of adolescents have been found to heighten the risk for poor mental health outcomes (e.g., non‐binary, minority status, low sense of ingroup identity) (Preston et al. 2025). These at‐risk groups include adolescents living in rural areas. Rural residents comprise 20% of the U.S. population and 16% of the Texas population (Hegar 2023; United States Census Bureau 2023b). An estimated 4.2 million Texans are considered rural and approximately 4.3 million Texans are adolescents (Brannen 2023; United States Census Bureau 2023a). Although an estimated number of rural Texas adolescents is not available, based on the known data it can be concluded that the rural adolescent population in Texas is substantial.

Rural children and adolescents have a higher prevalence of mental health disorders than their urban counterparts, with the ages of 11 to 14 being a time of increased risk for mental health problems (Crouch et al. 2023; Morales et al. 2020; Yoon et al. 2021). Some of the leading causes of illness among adolescents are depression, anxiety, and behavior disorders (Centers for Disease Control and Prevention 2024; World Health Organization 2025). While it has been found that females experience a greater decline in mental health during adolescence, adolescent males are less likely to seek mental health care (Rice et al. 2019; Yoon et al. 2023), possibly contributing to a gender discrepancy in the reported incidence of mental illness.

Adolescents, particularly rural adolescents, are in need of access to mental health care; however, rural Texas counties are facing a mental healthcare provider shortage (Health Resources and Services Administration 2025). This gap in mental health care impacts the entire rural community, including the adolescent population. The positive psychology approach nurturing strengths before crisis or mental health treatment is required may be an avenue to promote rural adolescent mental health. Very little is known about the intrinsic possession of positive psychological strengths (hope, self‐efficacy, resilience, optimism, and gratitude) among rural Texas adolescents. Additionally, since the announcement of the mental health crisis in 2021, research has lagged regarding how different genders perceive psychological strengths among adolescents living in the southern U.S. (U.S. Department of Health and Human Services, Office of the Surgeon General 2021; American Academy of Pediatrics 2021; The Trevor Project 2025). An understanding of unique gender differences in intrinsic psychological strengths among rural U.S. male, female, and non‐binary adolescents is needed in order to design tailored promotive mental health interventions (Mmari et al. 2024; Yoon et al. 2022).

### 1.4 Study Purpose

From an individual to a societal level, understanding positive psychological strengths has the potential to promote mental health among rural adolescents. Therefore, in this study we had a twofold purpose of exploring how adolescents self‐reported psychological strengths and examining gender differences in self‐reported psychological strengths. The associated research questions were:

1. How do rural Texas adolescents self‐report psychological strengths?
2. Are there group differences in positive psychological strengths among rural Texas adolescents?

## 2 Methods

### 2.1 Study Design, Sample, and Instruments

A secondary analysis was our selected method to address the proposed research questions. This analysis was deemed exempt by the primary investigator's university institutional review board. Study participants included 9th–12th grade students from one high school in rural Northeast Texas, an area with an overall higher‐than‐average teen pregnancy rate and juvenile detention rate in the state and in the U.S. The students were provided a QR code to the electronic survey packet, displayed on a white board by their teachers, during a regularly scheduled study hall period. The study was approved for opt‐out consent. Details concerning the study instruments are located in Table 1.

**Table 1:** Psychometric Properties of Measures of Psychological Strengths.

Psychological construct	# items and format	Benchmark mean*	Mean *(range)* SD	Cronbach's alpha	Sample item
Hope	6 items	25	23 *(6–36)*	0.86	*I think I am doing pretty well*.
Snyder et al. (1997)	6‐point Likert	*Bean* et al. *2020*	SD = 6.6		
Self‐efficacy *Schwarzer & Jerusalem 1995*	10 items	30	28 (*10–40)*	0.90	*If someone opposes me, I can find the means and ways to get what I want*.
	4‐point Likert	*Cramm* et al. *2012*	SD = 6.1		
Resilience Connor and Davidson 2003	10 items	32	24 *(0–40)*	0.89	*I am able to adapt when changes occur*.
	5‐point Likert	Connor and Davidson (2003)	SD = 8.2		
Optimism Scheier et al. 1994	6 items**	17	12 *(2–24)*	0.57	*In uncertain times, I usually expect the best*.
	5‐point Likert	Schou‐Bredal et al. (2017)	SD = 3.6		
McCullough et al. 2002	6 items	29	29 *(6–42)*	0.75	*I have so much in life to be thankful for*.
	7‐point Likert	Froh et al. (2011)	SD = 5.8		

*Note:* Benchmark mean set based on previously cited mean scores with population‐based norm or pediatric/adolescent population. Optimism scale contains 10 items but only 6 are scored (4 items are fillers).

### 2.2 Data Analysis

Basic rules of parametric analysis were followed, and missing values were handled using listwise deletion (Kang 2013; Polit and Beck 2017). Analyses were conducted on the RStudio platform (RStudio Team 2021). Measures of central tendency and psychometric properties were computed for all scales. The data was analyzed for extreme outliers but no responses were removed as no extreme outliers were identified (Polit and Beck 2017).

To establish benchmarks, the high benchmark and low benchmark scores were based on mean scores reported in previously established literature for each of the psychological constructs tested (Bean 2020; Cramm et al. 2013; Davidson 2025; Froh et al. 2011; Gilman et al. 2006; Jeevarajan et al. 2025; Li and Li 2020; Schou‐Bredal et al. 2017). Studies for benchmark setting were selected based on availability of data with a sample demographically‐similar to our sample population (e.g., U.S. adolescents). In some cases, no data was available with a sample demographically‐similar, so we utilized population‐based norms. The mean score for each participant in this study was calculated and attributed as greater than or equal to the group mean score (high benchmark) or less than the group mean score (low benchmark). This approach was chosen to improve both the rigor of our study and ability to compare findings.

Analysis of variance (ANOVA) testing was conducted to examine differences and effect sizes based on gender, race, and poverty status (factors we believed could impact development, or reporting, of an adolescent's psychological strengths). Post hoc tests were performed to examine significance of differences between groups following significant omnibus ANOVA results, including Tukey HSD and Bonferroni (Polit and Beck 2017).

## 3 Results

A total of 425 valid responses were retained. The sample was racially/ethnically diverse, with students identifying as 38.4% white (34.6% Hispanic, 15.3% Black, 3.5% Native American, and 1.4% Asian; 6.8% missing) and 51.1% female (42.4% male, 3.5% non‐binary; 3% missing). The mean age was 15.8 years (SD = 1.4 years). Over 74% of the sample lived in poverty and nearly 18% had a learning disability. Additional demographic details can be found in Table 2.

**Table 2:** Demographics of Rural Adolescent Sample (*N* = 425).

	Frequency *n*	Percent (%)
Racial Categories		
Black	65	15.3%
Hispanic	147	34.6%
Native American/Alaska Native	15	3.5%
Asian	6	1.4%
White	163	38.4%
Missing	29	6.8%
Gender Identity		
Male	180	42.4%
Female	217	51.1%
Non‐Binary	15	3.5%
Missing	13	3.0%
Living in poverty		
No Free lunch	24	5.7%
Free lunch	315	74.1%
Unsure	74	17.4%
Missing	12	2.8%
Age		
Mean	15.8 years	
Standard Deviation	1.4 years	
15 years and under		42.6%
16 years and over		48.7%
Missing	37	8.7%

*Note:* Demographics survey was placed at end of survey packet. All students participating were in grades 9–12.

### 3.1 Self‐ Reported Psychological Strengths

**Figure 1:**
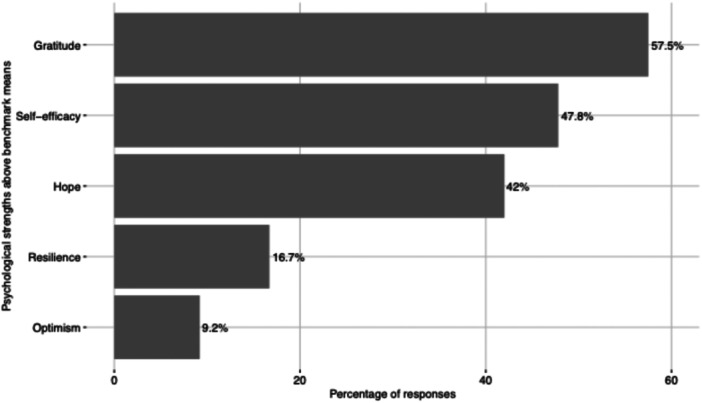
Self – reported psychological strengths above benchmark means.

To address the first research question, we analyzed self‐report scores of hope, self‐efficacy, resilience, optimism, and gratitude. Hope scores ranged from 6 to 36, with a mean score of 23 (SD = 6.6; median = 23) and 42% reported high levels of hope based on the established benchmark (Gilman et al. 2006). Self‐efficacy scores ranged from 10 to 40, with a mean score of 28 (SD = 6.1; median = 29) and 47.8% reported high levels of self‐efficacy (Cramm et al. 2013). Resilience scores ranged from 0 to 40, with a mean score of 24 (SD = 8.2; median = 24), and 16.7% reported high levels of resilience (Davidson 2025). Optimism scores ranged from 2 to 24, with a mean score of 12 (SD = 3.6; median = 11) and 9.2% reported high levels of optimism (Schou‐Bredal et al. 2017). Gratitude scores ranged from 6 to 42, with a mean score of 29 (SD = 5.8; median = 29) and 57.5% reported high levels of gratitude (Froh et al. 2011) (see Figure 1).

### 3.2 Demographic Differences in Psychological Strengths

We analyzed demographic differences in self‐report scores of hope, self‐efficacy, resilience, optimism, and gratitude to address our second research question. Significant differences were noted based on gender (see Table 3). Although not significant, the mean scores reported by Native American adolescents were lower than the other racial groups for the constructs hope, self‐efficacy, resilience, and optimism. Additionally, it should be noted that while the male and female groups are similar in number of respondents, there were fewer non‐binary respondents within this sample (male *n* = 180, female *n* = 217, non‐binary *n* = 15). Detailed results based on gender differences are provided in the next section.

**Table 3:** Differences in Psychological Strengths by Group.

Group	*n*	Hope Mean (SD)	*p*‐value	Self‐efficacy Mean (SD)	*p*‐value	Resilience Mean (SD)	*p*‐value	Optimism Mean (SD)	*p*‐value	Gratitude Mean (SD)	*p*‐value
Gender			0.000***		0.009**		0.003**		0.080		0.025*
Male	180	24.1 (7.14)		28.2 (7.29)		24.9 (9.38)		11.4 (4.51)		29.2 (6.58)	
Female	217	21.5 (7.14)		26.8 (7.28)		22.3 (9.38)		10.0 (4.51)		28.1 (6.57)	
Non‐Binary	15	16.9 (7.14)		22.9 (7.29)		18.5 (9.39)		8.0 (4.51)		24.9 (6.57)	
Race			0.236		0.067		0.248		0.799		0.207
Black	65	22.7 (7.37)		27.5 (7.28)		24.0 (9.36)		10.7 (4.43)		27.3 (6.31)	
Hispanic	147	23.1 (7.38)		28.4 (7.29)		24.1 (9.36)		10.9 (4.44)		28.6 (6.32)	
ANAI	15	19.0 (7.38)		23.3 (7.29)		18.4 (9.36)		10.1 (4.44)		30.1 (6.31)	
Asian	6	25.0 (7.37)		29.2 (7.29)		24.5 (9.36)		11.2 (4.44)		31.7 (6.31)	
White	163	22.0 (7.37)		26.8 (7.28)		23.2 (9.36)		10.5 (4.44)		29.0 (6.32)	
Poverty Status			0.662		0.972		0.807		0.099		0.405
Free Lunch	315	21.4 (7.32)		27.2 (7.37)		22.2 (9.52)		9.2 (4.51)		26.8 (6.61)	
No Free Lunch	24	22.5 (7.32)		27.3 (7.37)		23.4 (9.51)		10.8 (4.51)		28.7 (6.61)	
Uncertain	74	23.0 (7.31)		27.1 (7.37)		23.0 (9.51)		10.1 (4.51)		28.3 (6.61)	

*Note:* Some data is missing from demographic variables.

* *p* < 0.05;

** *p* < 0.01;

*** *p* < 0.001.

#### 3.2.1 Hope

There was a significant difference in hope between gender groups: (*F*(2, 409) = 10.78, *p* value = 0.000, η^2^ = 0.05). Once running post hoc tests, we found males scored significantly higher than females (mean difference estimate [Mdiff] = 2.51, *p* value = 0.001, 95% CI [1.09, 3.93]). We also found males scored significantly higher than non‐binary adolescents (Mdiff = 7.12, *p* value = 0.001, 95% CI [3.34, 10.90]). Females also scored significantly higher than non‐binary adolescents (Mdiff = 4.61, *p* value = 0.042, 95% CI [0.85, 8.37]).

#### 3.2.2 Self‐Efficacy

There was a significant difference in self‐efficacy between gender groups (*F*(2, 409) = 4.79, *p* value = 0.009, η^2^ = 0.02). Once running post hoc tests, we found males scored significantly higher than non‐binary adolescents (Mdiff = 5.31, *p* value = 0.019, 95% CI [1.46, 9.16]).

#### 3.2.3 Resilience

There was a significant difference in resilience between gender groups (F(2,409) = 5.89, *p* value = 0.003, η^2^ = 0.03). Once running post hoc tests, we found males scored significantly higher than female adolescents (Mdiff = 2.62, *p* value = 0.018, 95% CI [0.76, 4.48]). We also found males scored significantly higher than non‐binary adolescents (Mdiff = 6.43, *p* value = 0.034, 95% CI [1.46, 11.40]).

#### 3.2.4 Gratitude

**Figure 2:**
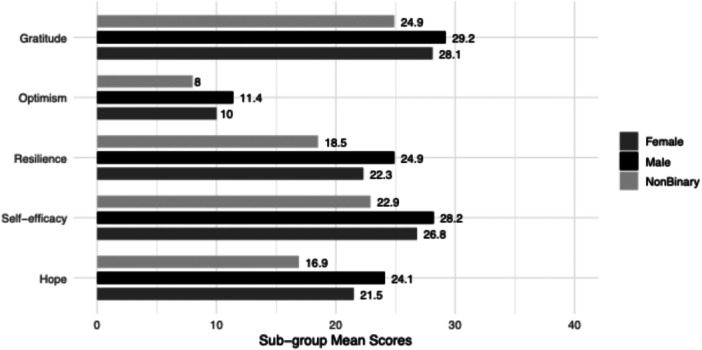
Gender group differences in mean reporting of psychological strengths. *Note*. Mean scores on psychological strengths are presented by gender group.

There was a significant difference in gratitude between gender groups (F(2,409) = 3.71, *p* value = 0.03, η^2^ = 0.02). Once running post hoc tests, we found males scored significantly higher than non‐binary adolescents (Mdiff = 4.31, *p* value = 0.04, 95% CI [0.83, 7.79]). See Figure 2 for comparison chart of positive psychological strengths between genders.

## 4 Discussion

In this study we sought to explore how adolescents report their psychological strengths, and to examine potential group differences at one rural high school in Texas. Overall, our findings demonstrated: (1) rural adolescents possess positive psychological strengths and (2) there are gender differences in reporting of psychological strengths. Males generally reported more positive psychological strengths than female and non‐binary adolescents.

### 4.1 Reporting of Psychological Strengths

One significant contribution of this study was that the majority of rural adolescents reported lower levels of optimism than any of the other psychological strengths examined (9.2% optimism). This finding was not entirely unexpected, when one considers the poor mental health among rural adolescents in this region of the state (Hirsch and Cukrowicz 2014; McCullumsmith et al. 2025) and the well‐known connection between optimism and mental health outcomes (Rincón Uribe et al. 2022). Several factors could be contributing to lower levels of optimism.

A longitudinal study of at‐risk Latinx youth from a low‐income community found developmental tasks to be associated with optimism scores (Taylor et al. 2020). Developmental tasks in the Taylor et al. (2020) study included the development of resilience. In our study, we found 16.7% of adolescents reported high levels of resilience (conversely, 83.3% reporting low levels of resilience), which aligns with this prior finding by Taylor et al. (2020), supporting the idea of a link between resilience and optimism. Taylor et al. (2020) also incorporated school connectedness and ethnic pride when construing adolescent developmental tasks. Aspects of the rural environment, such as school connectedness and ethnic pride, should be investigated as potential contributors to the development (or lack of development) of psychological strengths (including optimism) in rural settings.

In another study comparing adolescents who lived in urban and rural areas (Bhattacharyya and Molinari 2024) rurality was associated with low optimism scores, and adolescent female optimism has been closely linked to friend support, while adolescent male optimism has been linked to family support (Tusaie‐Mumford 2001). These findings have been replicated in work by Garandeau et al. (2022). The connection between optimism and depression, emotional problems, and substance use and other coping strategies (Patton et al. 2011; Puskar et al. 1999) warrants a closer look at: a) rural environmental factors which might impact the development of positive psychological strengths and b) how early screening might be used to identify adolescents at‐risk of low optimism (and poor mental health outcomes). While we did not find significant gender differences in optimism scores, we did find this sample of rural adolescents reported a lower mean score overall compared to the benchmark study, which was a population‐based norm and the closest available comparison sample (Schou‐Bredal et al. 2017).

### 4.2 Gender Differences in Psychological Strengths

The second significant contribution of this study was that, overall, males reported significantly greater levels of positive psychological strengths than females and non‐binary adolescents. Males scored significantly higher than females and non‐binary adolescents in both hope and resilience. While females scored significantly lower than males in self‐reported hope, they also scored significantly higher than non‐binary adolescents in hope. In a report presented by the University of Texas at Tyler School of Medicine, which utilized national datasets, such as CDC Wonder and the Youth Risk Behavior Surveillance Survey (YRBSS), they found female adolescents reported greater levels of hopelessness (57.2%) compared to hopelessness among male adolescents (32.1%) in Texas, both of which were slightly worse than the U.S. national average (McCullumsmith et al. 2025). We could find no other studies that have explored differences in hope between female and non‐binary adolescents. The closest available comparison (to our knowledge) is in the report prepared by McCullumsmith et al. (2025) where they presented findings in relation to sexual orientation and found that lesbian, gay, bisexual, and questioning adolescents reported up to 74.4% hopelessness as compared to 38.2% hopelessness reported by their heterosexual peers in Texas.

A study conducted with bullied high school students in Indonesia explored the concepts of hope and resilience, and found females' mean resilience scores were significantly higher than males' (Dewi et al. 2023). Considering male adolescents in our sample reported greater levels of resilience than females, we found this difference in male versus female resilience reporting conflicting. Several factors could contribute to differences between our study and the one by Dewi et al. (2023).

The study by Dewi et al. (2023) was conducted in the Far East and the current study was conducted in the rural southwest United States; however, data for both studies were collected following COVID‐19. Bullying, described as experiences of violence and rejection (Dewi et al. 2023), as well as ACEs, and the impact of how those experiences are perceived in the presence of psychological strengths should be further explored, particularly among rural Texas adolescents, considering they have also been found to report higher levels of experiencing or witnessing violence as compared to the national average (McCullumsmith et al. 2025).

While we could find one study that reported differing levels of resilience between transmasculine, transfeminine, and non‐binary adolescents and young adults (AYA) (Poquiz et al. 2021), we could find no studies currently available that reported on gender differences in resilience between binary (i.e., identify as male or female) and non‐binary adolescents. The closest comparison available was a study conducted in rural Texas examining psychological capital (combined *HERO* strengths) and mental health. Non‐binary adolescents reported significantly lower psychological capital than their binary peers (Preston et al. 2025).

One last important note about resilience that we found interesting is that within our sample of rural Texas adolescents, the mean score was 24. To set the benchmark, the closest sample we could find from the general population was from the scale developer website, which included adolescents and adults. The mean from this general population sample was 32 (Davidson 2025); however, we did find one other study recently conducted with Texas youth who currently were receiving psychiatric health services or had screened positive for depression. Within this sample of youth receiving psychiatric services (*n* = 908), their mean resilience score was 19 (Jeevarajan et al. 2025). Our study adds important knowledge concerning the mean score for a general population U.S. rural adolescent sample. While the adolescents in our study had overall low resilience compared to population‐based data (only 16.7% scored as having “high” resilience), their mean score was higher than the psychiatric sample from Jeevarajan et al. (2025). This adds to the knowledge base concerning the protective effect of resilience and its potential to delay the requirement for psychiatric services, and raises the question concerning what other contributing factors might be involved that alter the trajectory from *being resilient in the community* to *no longer resilient and need crisis intervention* (as evidenced through the requirement of psychiatric services).

Going back to a theoretical understanding of resilience from the field of positive organizational behavior, resilience is the product of three circumstances, the existence of assets, risks factors, and an underlying value system (Luthans et al. 2007). Under the assumption that the underlying value system is constant, the altered trajectory could be attributed to asset depletion or increase in risk factors. Furthermore, assets include social support, psychological strengths such as hope and optimism, and tangible resources (Luthans et al. 2007). Risk factors include any combination of risks including substance use exposure, violence, and other ACEs.

The sample in this study had similar levels of hope, self‐efficacy, and gratitude as the benchmark samples, yet levels of optimism were very low (88.8% in this study reported a lower mean score than the population‐ based norm). Additionally, we know assets (like access to mental health care in this geographic area) are critically low (McCullumsmith et al. 2025). It is necessary for adults who interact with rural adolescents in under‐resourced areas to take measures to amplify their psychological strengths and develop infrastructure that can serve as assets for the development of resilience.

Although we found no gender differences in optimism, prior research conducted with rural Pennsylvanian high school students found male adolescents scored significantly higher than female adolescents in both self‐esteem and optimism (Puskar et al. 2010). Optimism scores were remarkably low within our sample, so further research is warranted to further investigate gender‐based outcomes as they may have been indiscernible concerning the overall low scores across all groups, which may indicate an even more urgent need for intervention, since male adolescents typically tend to report higher levels of psychological strengths than other gender groups (Katz et al. 2019).

Lastly, rural male adolescents reported greater self‐efficacy and gratitude than rural non‐binary adolescents. No prior literature was available to compare this finding to previous studies. Tankersley et al. (2021) conducted a literature review which highlighted the importance of resilience‐promoting factors among non‐binary youth, so we found it surprising that little work has been done to expand knowledge in this area in relation to psychological strengths and rural youth at heightened risk for poor mental health outcomes, such as those reporting non‐binary identity. This study fills a gap in the literature concerning gender differences in psychological strengths.

### 4.3 Practice Implications

Nurses working with adolescents at the individual and population level (e.g., public health, schools) should partner with community agencies to work together to promote positive development of psychological strengths. Adolescents engage with society on a micro‐ and macro‐level through local school activities, in peer‐to‐peer interactions, and on social media outlets that reach a wide platform. Nurses and other healthcare professionals should educate on the benefits of developing psychological strengths, and support initiatives that foster optimistic mindsets. When considering interventions to promote adolescent mental health, the World Health Organization recommends two key principles, one of which is a focus on the interconnection of services including non‐health settings such as schools, youth centers, and digital technologies (World Health Organization & United Nations Fund 2024). The American Psychological Association Summit placed an urgent call for practitioners to focus on population level, culturally‐tailored care approaches that promote resilience in communities and to incorporate universal screenings (Dodge et al. 2024). For both in‐person and digitized interventions, tailoring with personalized content and peer engagement have been found to enhance treatment outcomes and user experience (Li et al. 2024; Opie et al. 2024). Nurses should incorporate these recommended principles and exercise connective caring to facilitate strengths development (Preston 2024).

The effect size of differences in psychological strengths between gender groups was small, accounting for 2%–5% of differences in *HERO* strengths and gratitude. While these differences are considered to be a small effect, the science in psychological strengths has not advanced enough for us to know how meaningful even the smallest increases in these strengths may impact behavioral health and decision‐making among adolescents. Practice interventions should target adolescent groups at‐risk of poor mental health outcomes, as well as parent‐like figures and schools, in educational and practice activities. Traumatic experiences and other ACEs are prevalent in society, so it is important that adolescents are equipped to weather life's storms. Psychological strengths are amenable to development and intervention to promote health (Luthans et al. 2007).

### 4.4 Strengths

A strength of this study is that it was conducted with a diverse sample of an often understudied and underserved group‐‐ rural adolescents. Another strength of this study was that we took into consideration demographic factors, such as gender, poverty, and race, and our findings demonstrated that among all groups, female and non‐binary gender identities were the most likely to report lower psychological strengths.

There was a strong choice of measures in this original study (Preston et al. 2025) which made it possible that we could compare the mean scores reported in this sample and compare our findings with other studies (Taylor et al. 2020).

### 4.5 Limitations

As with all secondary analyses, we were limited by the variables tested in the original study and the order in which measures were presented. As such, we could not examine mental health outcomes such as depression or anxiety, as that data was not originally collected. We also noted 2.8% – 8.7% missingness for demographic items; these items were placed at the end of the survey packet when the data was originally collected. Furthermore, seeing as this was cross‐sectional, there is the possibility that scores reported by the adolescents participating in this study might have fluctuated from day to day, and there could be potential differences between those who chose to complete the questionnaire and those who did not, which highlights the benefit of examining reporting of psychological strengths over time through longitudinal research.

The data was collected at one school district in rural Texas, limiting generalizability of the findings; however limited, this area of Texas has higher‐than‐average suicide rates, teen pregnancy rates, and juvenile detention rates, so the findings are insightful in relation to health outcomes in this area (Texas Department of State Health Services 2024; Texas Juvenile Justice Department 2023; The Sentencing Project 2025). Lastly, there were 15 participants who identified as non‐binary (compared to 180 who identified as male and 217 who identified as female), so comparisons involving non‐binary adolescents should be interpreted with caution. We believe it is an important contribution to acknowledge the existence of non‐binary adolescents living and attending school in a rural area; regions which are typically known to be culturally‐against non‐binary gender identification, with limited acknowledgement of the existence of these adolescents in their community (The Trevor Project 2021).

Additionally, we set our benchmark to distinguish between high versus low scores using previously cited literature, which is considered a robust approach and a commonly accepted practice. While there are limitations, when no established cutoff or normative data exists for a questionnaire for a particular population, this strategy is less sensitive to outliers than using the mean and makes findings more comparable across studies. Still, this method is sample‐dependent and the cutoff is relative to what limited literature exists concerning psychological strengths and the adolescent population. As an example, the population‐based norm for the benchmark mean for optimism was based on a sample of Norwegian adults, because it was the only population‐based norm available. It is our belief that more population‐based norms are needed, as from what we could find, it appeared overall mean scores for adult populations appear to be higher than adolescent populations, which may indicate attainment of psychological strengths is indeed a developmental task of adolescence and requires more investigation.

Furthermore, in our discussion of resilience scores, some of our interpretations may be culturally confounded, because we had to interpret scores by comparing our findings with previous studies conducted with adolescents outside of the U.S. (such as Indonesian adolescents). A larger body of work on adolescents' psychological strengths has been conducted outside of the U.S. than what has been conducted within the U.S.

## 5 Conclusion

Our aim to explore the self‐reported psychological strengths and to examine gender differences was met. Despite limitations, health disparities exist within this rural sample, making it an important group to study and further support. Promotive healthcare measures not only focused on the physical well‐being, but also the mental well‐being, of future generations of adults is of importance to all involved and close consideration for developing psychological strengths in rural, under‐resourced regions is needed.

## 6 How Might This Information Affect Practice?

Females and non‐binary adolescents are at heightened risk for lower reporting of positive psychological strengths. School professionals should work with stakeholders, across clinic, school, and other community settings, to consider how to promote psychological strengths, considering not all adolescents possess the same levels of these inner strengths. Prior studies have demonstrated psychological strengths are linked to overall wellbeing and also performance outcomes, such as work and school performance. Tailored programming that considers additional environmental risk factors, such as bullying experiences and ACEs, should be incorporated to expand promotive service criteria for rural adolescents.

## Author Contributions

All authors contributed to conceptualization. Angela J. Preston conducted analysis. Angela J. Preston wrote original manuscript in conjunction with Jodi Bullard, Rachel Smith, and Ashlyn Peppler. Final review was conducted by Angela J. Preston and Jodi Bullard.

## Funding

This research was funded in part by a dissertation grant to the first author by Jonas Philanthropies and Sigma Theta Tau International Iota Nu Chapter. The expressions contained within are the not representing these organizations and are solely those of the authors.

## Ethics Statement

This secondary analysis was deemed IRB exempt. All requirements of the Declaration of Helsinki were followed.

## Conflicts of Interest

The authors declare no conflicts of interest.

## Data Availability

Data is maintained solely by the corresponding author and available upon request due to privacy reasons.

## References

[jcap70048-bib-0001] Agency for Healthcare Research and Quality . 2022. Child and Adolescent Mental Health. https://www.ncbi.nlm.nih.gov/books/NBK587174/.

[jcap70048-bib-0003] American Academy of Pediatrics . 2021. AAP‐AACAP‐CHA Declaration of a National Emergency in Child and Adolescent Mental Health. https://www.aap.org/en/advocacy/child-and-adolescent-healthy-mental-development/aap-aacap-cha-declaration-of-a-national-emergency-in-child-and-adolescent-mental-health/?srsltid=AfmBOorB1lRBXen7jtY9VhwS0Mo_B2-c4HYopwEWX2_MtSUDjU8_qocG.

[jcap70048-bib-0004] Barry, M. M. , T. Kuosmanen , T. Keppler , K. Dowling , and P. Harte . 2024. “Priority Actions for Promoting Population Mental Health and Wellbeing.” Mental Health & Prevention 33: 200312. 10.1016/j.mhp.2023.200312.

[jcap70048-bib-0005] Bean, G. J. 2020. “An Item Response Theory Analysis of the Children's Hope Scale.” Journal of the Society for Social Work and Research 11, no. 2: 339–364. 10.1086/709455.

[jcap70048-bib-0006] Best, O. , and S. Ban . 2021. “Adolescence: Physical Changes and Neurological Development.” British Journal of Nursing 30, no. 5: 272–275. 10.12968/bjon.2021.30.5.272. 33733842

[jcap70048-bib-0007] Bhattacharyya, K. K. , and V. Molinari . 2024. “Impact of Optimism on Cognitive Performance of People Living in Rural Area: Findings From a 20‐Year Study in US Adults.” Gerontology and Geriatric Medicine 10: 23337214241239147. 10.1177/23337214241239147. 38500788 PMC10946068

[jcap70048-bib-0008] Brannen, J. 2023. *After Census Redefines Urban and Rural, Texas Remains Steadfastly Both*. https://kinder.rice.edu/urbanedge/census-redefines-urban-rural.

[jcap70048-bib-0009] Centers for Disease Control and Prevention . 2024. *Child Mental Health: Rural Policy Brief*. https://www.cdc.gov/rural-health/php/policy-briefs/child-mental-health-policy-brief.html.

[jcap70048-bib-0010] Colizzi, M. , A. Lasalvia , and M. Ruggeri . 2020. “Prevention and Early Intervention in Youth Mental Health: Is it Time for a Multidisciplinary and Trans‐Diagnostic Model for Care?” International Journal of Mental Health Systems 14, no. 1: 23. 10.1186/s13033-020-00356-9. 32226481 PMC7092613

[jcap70048-bib-0011] Connor, K. M. , and J. R. T. Davidson . 2003. “Development of a New Resilience Scale: The Connor‐Davidson Resilience Scale (CD‐RISC).” Depression and Anxiety 18, no. 2: 76–82. 10.1002/da.10113. 12964174

[jcap70048-bib-0012] Cramm, J. M. , M. M. H. Strating , M. E. Roebroeck , and A. P. Nieboer . 2013. “The Importance of General Self‐Efficacy for the Quality of Life of Adolescents with Chronic Conditions.” Social Indicators Research 113, no. 1: 551–561. 10.1007/s11205-012-0110-0. 23874059 PMC3696170

[jcap70048-bib-0080] Crouch, E. , P. Hung , G. Benavidez , T. Giannouchos , and M. J. Brown . 2023. “Rural‐urban Differences in Access to Care Among Children and Adolescents in the United States.” Journal of Rural Health 40, 1: 200–207. 10.1111/jrh.12769. 37217438

[jcap70048-bib-0014] Davidson, J. R. 2025. *The Connor‐Davidson Resilience Scale*. https://www.connordavidson-resiliencescale.com/about.php.

[jcap70048-bib-0015] Dewi, N. S. , M. Andriany , O. I. Lathifah , et al. 2023. “The Influence of Gender and Hope on the Resilience of Bullied Adolescents.” Journal Keperawatan Indonesia 26, no. 1: 57–67. 10.7454/jki.v26i1.1784.

[jcap70048-bib-0016] Dodge, K. A. , M. J. Prinstein , A. C. Evans , et al. 2024. “Population Mental Health Science: Guiding Principles and Initial Agenda.” American Psychologist 79, no. 6: 805–823. 10.1037/amp0001334. 38829360

[jcap70048-bib-0017] Finch, J. , A. M. Waters , and L. J. Farrell . 2023. “Developing the HERO within: Evaluation of a Brief Intervention for Increasing Psychological Capital (PsyCap) in Australian Female Students During the Final Year of School in the First Year of COVID‐19.” Journal of Affective Disorders 324: 616–623. 10.1016/j.jad.2022.12.169. 36621678 PMC9814284

[jcap70048-bib-0018] Froh, J. J. , J. Fan , R. A. Emmons , G. Bono , E. S. Huebner , and P. Watkins . 2011. “Measuring Gratitude in Youth: Assessing the Psychometric Properties of Adult Gratitude Scales in Children and Adolescents.” Psychological Assessment 23, no. 2: 311–324. 10.1037/a0021590. 21443367

[jcap70048-bib-0019] Gambin, M. , and M. Święcicka . 2012. “Construction and Validation of Self‐Efficacy Scale for Early School‐Aged Children.” European Journal of Developmental Psychology 9: 723–729. 10.1080/17405629.2012.688100.

[jcap70048-bib-0020] Garandeau, C. F. , L. Laninga‐Wijnen , and C. Salmivalli . 2022. “Effects of the KiVa Anti‐Bullying Program on Affective and Cognitive Empathy in Children and Adolescents.” Journal of Clinical Child and Adolescent Psychology: The Official Journal for the Society of Clinical Child and Adolescent Psychology, American Psychological Association, Division 53 51, no. 4: 515–529. 10.1080/15374416.2020.1846541. 33448897

[jcap70048-bib-0021] Gilman, R. , J. Dooley , and D. Florell . 2006. “Relative Levels of Hope and Their Relationship With Academic and Psychological Indicators Among Adolescents.” Journal of Social and Clinical Psychology 25, no. 2: 166–178. 10.1521/jscp.2006.25.2.166.

[jcap70048-bib-0022] Health Resources and Services Administration . 2025. HPSA find. https://data.hrsa.gov/tools/shortage-area/hpsa-find.

[jcap70048-bib-0023] Hegar, G. 2023. Defining Rural Texas: Identifying and Supporting Rural Areas. https://comptroller.texas.gov/economy/fiscal-notes/archive/2023/aug/rural.php.

[jcap70048-bib-0024] Hinojosa, M. S. , and R. Hinojosa . 2024. “Positive and Adverse Childhood Experiences and Mental Health Outcomes of Children.” Child Abuse & Neglect 149: 106603. 10.1016/j.chiabu.2023.106603. 38141478

[jcap70048-bib-0025] Hirsch, J. K. , and K. C. Cukrowicz . 2014. “Suicide in Rural Areas: An Updated Review of the Literature.” Journal of Rural Mental Health 38, no. 2: 65–78. 10.1037/rmh0000018.

[jcap70048-bib-0026] Hughes, K. , M. A. Bellis , K. A. Hardcastle , et al. 2017. “The Effect of Multiple Adverse Childhood Experiences on Health: A Systematic Review and Meta‐Analysis.” Lancet Public Health 2, no. 8: e356–e366. 10.1016/s2468-2667(17)30118-4. 29253477

[jcap70048-bib-0027] Jeevarajan, J. R. , A. Theodorou , K. Nandy , et al. 2025. “Psychometric Properties of the 10‐item Connor‐Davidson Resilience Scale (CD‐RISC‐10) in Adolescent and Young Adult Psychiatric Outpatients in the Texas Youth Depression and Suicide Research Network (TX‐YDSRN).” Journal of Affective Disorders 375: 155–164. 10.1016/j.jad.2025.01.091. 39848473

[jcap70048-bib-0028] Kang, H. 2013. “The Prevention and Handling of the Missing Data.” Korean Journal of Anesthesiology 64, no. 5: 402–406. https://pubmed.ncbi.nlm.nih.gov/23741561/. 23741561 10.4097/kjae.2013.64.5.402 PMC3668100

[jcap70048-bib-0029] Katz, I. T. , L. M. Bogart , J. J. Dietrich , et al. 2019. “Understanding the Role of Resilience Resources, Antiretroviral Therapy Initiation, and HIV‐1 RNA Suppression Among People Living With HIV in South Africa: A Prospective Cohort Study.” Supplement, AIDS 33, no. Suppl 1: S71–S79. 10.1097/qad.0000000000002175. 31397725 PMC6712569

[jcap70048-bib-0030] Kayser, V. , and I. Ramzan . 2021. “Vaccines and Vaccination: History and Emerging Issues.” Human Vaccines & Immunotherapeutics 17, no. 12: 5255–5268. 10.1080/21645515.2021.1977057. 34582315 PMC8903967

[jcap70048-bib-0031] Li, L. , and M. Li . 2020. “Effects of Mindfulness Training on Psychological Capital, Depression, and Procrastination of the Youth Demographic.” Iranian Journal of Public Health 49, no. 9: 1692–1700. 10.18502/ijph.v49i9.4086. 33643944 PMC7898108

[jcap70048-bib-0032] Li, W. , J. Gleeson , and M. I. Fraser , et al. 2024. “The Efficacy of Personalized Psychological Interventions in Adolescents: A Scoping Review and Meta‐Analysis.” Frontiers in Psychology 15: 1470817. 10.3389/fpsyg.2024.1470817. 39309145 PMC11413809

[jcap70048-bib-0033] Luthans, F. , C. M. Youssef , and B. J. Avolio . 2007. Psychological Capital: Developing the Human Competitive Edge. Oxford university press.

[jcap70048-bib-0079] McCullough, M. E. , R. A. Emmons , and J.‐A. Tsang . 2002. “The Grateful Disposition: A Conceptual and Empirical Topography.” Journal of Personality and Social Psychology 82, no. 1: 112–127. 10.1037/0022-3514.82.1.112. 11811629

[jcap70048-bib-0034] McCullumsmith, C. , S. McBride , C. Alvarado , et al. 2025. The Status of Mental Health in Northeast Texas 2024 . [Unpublished].

[jcap70048-bib-0035] McGorry, P. D. , and C. Mei . 2018. “Early Intervention in Youth Mental Health: Progress and Future Directions.” Evidence Based Mental Health 21, no. 4: 182–184. 10.1136/ebmental-2018-300060. 30352884 PMC10270418

[jcap70048-bib-0036] Mmari, K. , C. Simon , and R. Verma . 2024. “Gender‐Transformative Interventions for Young Adolescents: What Have We Learned and Where Should We go?” Supplement, Journal of Adolescent Health 75, no. 4, Suppl: S62–S80. 10.1016/j.jadohealth.2024.04.016. 39293879

[jcap70048-bib-0037] Morales, D. A. , C. L. Barksdale , and A. C. Beckel‐Mitchener . 2020. “A Call to Action to Address Rural Mental Health Disparities.” Journal of Clinical and Translational Science 4, no. 5: 463–467. https://pubmed.ncbi.nlm.nih.gov/33244437/. 33244437 10.1017/cts.2020.42 PMC7681156

[jcap70048-bib-0038] National Alliance on Mental Illness (n.d.). *Mental Health in Schools*. https://www.nami.org/Advocacy/Policy-Priorities/Improving-Health/Mental-Health-in-Schools.

[jcap70048-bib-0039] National Alliance on Mental Illness . 2024. Mental Health in Numbers. https://www.nami.org/about-mental-illness/mental-health-by-the-numbers/.

[jcap70048-bib-0040] National Institute of Mental Health . 2024. *Mental illness*. https://www.nimh.nih.gov/health/statistics/mental-illness.

[jcap70048-bib-0041] Opie, J. E. , A. Vuong , E. T. Welsh , T. B. Esler , U. R. Khan , and H. Khalil . 2024. “Outcomes of Best‐Practice Guided Digital Mental Health Interventions for Youth and Young Adults with Emerging Symptoms: Part II. A Systematic Review of User Experience Outcomes.” Clinical Child and Family Psychology Review 27, no. 2: 476–508. 10.1007/s10567-024-00469-4. 38634939 PMC11222193

[jcap70048-bib-0042] Ortiz, R. 2019. “Building Resilience Against the Sequelae of Adverse Childhood Experiences: Rise up, Change Your Life, and Reform Health Care.” American Journal of Lifestyle Medicine 13, no. 5: 470–479. 10.1177/1559827619839997. 31523212 PMC6732880

[jcap70048-bib-0043] Patton, G. C. , M. M. Tollit , H. Romaniuk , S. H. Spence , J. Sheffield , and M. G. Sawyer . 2011. “A Prospective Study of the Effects of Optimism on Adolescent Health Risks.” Pediatrics 127, no. 2: 308–316. 10.1542/peds.2010-0748. 21220404

[jcap70048-bib-0044] Polit, D. F. , and C. T. Beck . 2017. Nursing Research: Generating and Assessing Evidence for Nursing Practice, 11 ed. Wolters Klower.

[jcap70048-bib-0045] Poquiz, J. L. , C. A. Coyne , R. Garofalo , and D. Chen . 2021. “Comparison of Gender Minority Stress and Resilience Among Transmasculine, Transfeminine, and Nonbinary Adolescents and Young Adults.” Journal of Adolescent Health 68, no. 3: 615–618. 10.1016/j.jadohealth.2020.06.014. PMC8479649 33046360

[jcap70048-bib-0046] Preston, A. , L. Rew , and C. Young . 2025. “Exploring Stigma and Mental Health: Patient‐Reported Outcomes Among Rural, Underserved Youth.” Advances in Patient‐ Reported Outcomes 1, no. 1: 100019. 10.1016/j.apro.2025.100019.

[jcap70048-bib-0047] Preston, A. , L. Rew , and C. C. Young . 2023. “A Systematic Scoping Review of Psychological Capital Related to Mental Health in Youth.” Journal of School Nursing 39, no. 1: 72–86. 10.1177/10598405211060415. 34898323

[jcap70048-bib-0048] Preston, A. J. 2024. “The Importance of Nurse–Patient Relationships in the Midst of Substance Misuse: Support for Rural Youth Well‐Being.” Journal of Psychosocial Nursing and Mental Health Services 62: 7–11. 10.3928/02793695-20240423-01. 38838338

[jcap70048-bib-0049] Preston, A. J. , K. Zhang , C. Young , and L. Rew 2025. *Decision‐making in Factor Analysis: PPQ‐E Psychometric Properties for Adolescent Health Research* [Manuscript submitted for publication]. School of Nursing, University of Texas at Austin.

[jcap70048-bib-0050] Puskar, K. R. , S. M. Sereika , J. Lamb , K. Tusaie‐Mumford , and T. McGuinness . 1999. “Optimism and Its Relationship to Depression, Coping, Anger, and Life Events in Rural Adolescents.” Issues in Mental Health Nursing 20, no. 2: 115–130. 10.1080/016128499248709. 10409992

[jcap70048-bib-0051] Puskar, K. R. , L. Marie Bernardo , D. Ren , et al. 2010. “Self‐Esteem and Optimism in Rural Youth: Gender Differences.” Contemporary Nurse 34, no. 2: 190–198. 10.5172/conu.2010.34.2.190. 20509803

[jcap70048-bib-0053] Rice, F. , L. Riglin , A. K. Thapar , et al. 2019. “Characterizing Developmental Trajectories and the Role of Neuropsychiatric Genetic Risk Variants in Early‐Onset Depression.” JAMA Psychiatry 76, no. 3: 306–313. 10.1001/jamapsychiatry.2018.3338. 30326013 PMC6439821

[jcap70048-bib-0054] Rincón Uribe, F. A. , C. A. Neira Espejo , and J. S. Pedroso . 2022. “The Role of Optimism in Adolescent Mental Health: A Systematic Review.” Journal of happiness studies 23, no. 2: 815–845. 10.1007/s10902-021-00425-x.

[jcap70048-bib-0055] RStudio Team . 2021. *RStudio: Integrated Development Environment for R*. RStudio. PBC. http://www.rstudio.com/.

[jcap70048-bib-0056] Scheier, M. F. , C. S. Carver , and M. W. Bridges . 1994. “Distinguishing Optimism From Neuroticism (And Trait Anxiety, Self‐Mastery, and Self‐Esteem): a Reevaluation of the Life Orientation Test.” Journal of Personality and Social Psychology 67, no. 6: 1063–1078. 10.1037//0022-3514.67.6.1063. 7815302

[jcap70048-bib-0057] Schou‐Bredal, I. , T. Heir , L. Skogstad , et al. 2017. “Population‐Based Norms of the Life Orientation Test‐Revised (LOT‐R).” International Journal of Clinical and Health Psychology 17, no. 3: 216–224. 10.1016/j.ijchp.2017.07.005. 30487897 PMC6220921

[jcap70048-bib-0058] Seligman, M. E. 2002. Handbook of Positive Psychology, edited by C. R. Snyder and S. J. Lopez . Oxford University Press.

[jcap70048-bib-0059] Singh, V. , A. Kumar , and S. Gupta . 2022. “Mental Health Prevention and Promotion—A Narrative Review.” Frontiers in Psychiatry 13: 898009. 10.3389/fpsyt.2022.898009. 35958637 PMC9360426

[jcap70048-bib-0060] Snyder, C. R. , B. Hoza , W. E. Pelham , et al. 1997. “The Development and Validation of the Children's Hope Scale.” Journal of Pediatric Psychology 22, no. 3: 399–421. 10.1093/jpepsy/22.3.399. 9212556

[jcap70048-bib-0061] Tankersley, A. P. , E. L. Grafsky , J. Dike , and R. T. Jones . 2021. “Risk and Resilience Factors for Mental Health Among Transgender and Gender Nonconforming (TGNC) Youth: A Systematic Review.” Clinical Child and Family Psychology Review 24, no. 2: 183–206. 10.1007/s10567-021-00344-6. 33594611

[jcap70048-bib-0062] Taylor, Z. E. , N. Kittrell , N. Nair , C. D. Evich , and B. L. Jones . 2020. “Developmental Antecedents of Adolescent Optimism in Rural Midwestern US Latinx Youth.” Journal of Community Psychology 48, no. 2: 448–463. 10.1002/jcop.22267. 31654590

[jcap70048-bib-0063] Texas Juvenile Justice Department . 2023. The State of Juvenile Probation Activity in Texas. Texas Juvenile Justice Department. https://www.tjjd.texas.gov/wp-content/uploads/2024/08/The-State-of-Juvenile-Probation-Activity-in-Texas-Calendar-Year-2023.pdf.

[jcap70048-bib-0064] The Sentencing Project . 2025. U.S. Criminal Justice Data. https://www.sentencingproject.org/research/us-criminal-justice-data/.

[jcap70048-bib-0065] The Trevor Project . 2021. LGBTQ Youth in Small Towns and Rural Areas. https://www.thetrevorproject.org/research-briefs/lgbtq-youth-in-small-towns-and-rural-areas/.

[jcap70048-bib-0066] The Trevor Project . 2025. The Mental Health and Experiences of LGBTQ+ Young People in the Rural U.S. 10.70226/XAGT4119.

[jcap70048-bib-0067] Tusaie‐Mumford, K. R. 2001. Psychosocial Resilience in Rural Adolescents: Optimism, Perceived Social Support and Gender Differences. University of Pittsburgh.

[jcap70048-bib-0068] United States Census Bureau, P. D. 2017. Projected 5‐year Age Groups and Sex Composition: Main Projections Series for the United States, 2017‐2060. https://www2.census.gov/programs-surveys/popproj/tables/2017/2017-summary-tables/np2017-t3.xlsx.

[jcap70048-bib-0069] United States Census Bureau, P. D. 2020. Current Population Survey, Annual Social and Economic supplement, 2019. https://www2.census.gov/programs-surveys/demo/tables/age-and-sex/2019/age-sex-composition/2019gender_Table1.xlsx.

[jcap70048-bib-0070] United States Census Bureau, P. D. 2023a. 2020 Census Urban Areas Facts. https://www.census.gov/programs-surveys/geography/guidance/geo-areas/urban-rural/2020-ua-facts.html.

[jcap70048-bib-0071] United States Census Bureau, P. D. 2023b. American Community Survey 2023: 5‐year Estimates Subject Tables. https://data.census.gov/table/ACSST5Y2023.S0101?g=040XX00US48&tid=ACSST5Y2023.S0101.

[jcap70048-bib-0072] U.S. Department of Health and Human Services, Office of the Surgeon General . 2021. “Protecting Youth Mental Health: The U.S.” Surgeon General's advisory. U.S. Department of Health and Human Services,1–53. https://www.hhs.gov/sites/default/files/surgeon-general-youth-mental-health-advisory.pdf.

[jcap70048-bib-0073] World Health Organization . 2024. Mental Health of Adolescents. https://www.who.int/news-room/fact-sheets/detail/adolescent-mental-health.

[jcap70048-bib-0074] World Health Organization . 2025. Adolescent Health. https://www.who.int/health-topics/adolescent-health#tab=tab_1.

[jcap70048-bib-0075] World Health Organization, & United Nations Fund . 2024. Mental Health of Children and Young People: Service Guidance. World Health Organization. https://www.who.int/publications/i/item/9789240100374.

[jcap70048-bib-0076] Texas Department of State Health Services . 2024. “Birth Demographics.” Texas Department of State Health Services, 1–53. https://www.tjjd.texas.gov/wp-content/uploads/2024/08/The-State-of-Juvenile-Probation-Activity-in-Texas-Calendar-Year-2023.pdf.

[jcap70048-bib-0077] Yoon, S. , K. Howell , R. Dillard , K. Shockley McCarthy , T. Rae Napier , and F. Pei . 2021. “Resilience Following Child Maltreatment: Definitional Considerations and Developmental Variations.” Trauma, violence & abuse 22, no. 3: 541–559. 10.1177/1524838019869094. 31405362

[jcap70048-bib-0078] Yoon, Y. , M. Eisenstadt , S. T. Lereya , and J. Deighton . 2023. “Gender Difference in the Change of Adolescents' Mental Health and Subjective Wellbeing Trajectories.” European Child & Adolescent Psychiatry 32, no. 9: 1569–1578. 10.1007/s00787-022-01961-4. 35246720 PMC8896070

